# ZIF-90 nanoparticles modified with a homing peptide for targeted delivery of cisplatin

**DOI:** 10.3389/fchem.2022.1076350

**Published:** 2022-12-05

**Authors:** Adamu Abubakar, Emilia Abdulmalek, Wan Norhamidah Wan Ibrahim, Kyle E. Cordova, Mohd Basyaruddin Abdul Rahman

**Affiliations:** ^1^ Integrated Chemical BioPhysics Research, Faculty of Science, Universiti Putra Malaysia (UPM), Seri Kembangan, Malaysia; ^2^ Department of Chemical Sciences, Taraba State University, P.M.B, Taraba State, Jalingo, Nigeria; ^3^ Department of Biology, Faculty of Science, UPM, Selangor Darul Ehsan, Malaysia; ^4^ Materials Discovery Research Unit, Advanced Research Centre, Royal Scientific Society, Amman, Jordan; ^5^ Department of Chemistry, Faculty of Science, UPM, Selangor Darul Ehsan, Malaysia; ^6^ Foundry of Reticular Materials for Sustainability (FORMS), Materials Synthesis and Characterization Laboratory, Institute of Advanced Technology, UPM, Selangor Darul Ehsan, Malaysia

**Keywords:** nanocarrier (nanoparticle), zeolitic imidalozate framework, post-synthetic modification (PSM), RGD, peptide, targeted delivery

## Abstract

To improve the selective delivery of cisplatin (Cis) to cancer cells, we report and establish the significance of active, targeting drug delivery nanosystems for efficient treatment of lung cancer. Specifically, pH-responsive nano-sized zeolitic imidazolate framework (nZIF-90) was synthesized, post-synthetically modified with an Arg-Gly-Asp peptide motif (RGD@nZIF-90), a known cancer cell homing peptide, and loaded with a large amount of Cis (RGD@Cis⊂nZIF-90). RGD@Cis⊂nZIF-90 was shown to be highly stable under physiological conditions (pH = 7.4) with framework dissociation occurring under slightly acidic conditions (pH = 5.0)–conditions relevant to tumor cells–from which 90% of the encapsulated Cis was released in a sustained manner. *In vitro* assays demonstrated that RGD@Cis⊂nZIF-90 achieved significantly better cytotoxicity (65% at 6.25 μg ml^−1^) and selectivity (selectivity index = 4.18 after 48 h of treatment) against adenocarcinoma alveolar epithelial cancer cells (A549) when compared with the unmodified Cis⊂nZIF-90 (22%). Cellular uptake using A549 cells indicated that RGD@Cis⊂nZIF-90 was rapidly internalized leading to significant cell death. After successfully realizing this nanocarrier system, we demonstrated its efficacy in transporting and delivering Cis to cancer cells.

## Introduction

Despite recent developments in photothermal and immune-based cancer therapy, conventional chemotherapy remains the primary approach for cancer treatment. (Chen et al., 2016; Flemming, 2016) Cisplatin (Cis; [cis-Pt (NH_3_)_2_(Cl)_2_]) is a highly effective, conventional antitumor chemotherapeutic drug that forms intra- and inter-strand cross-links with DNA leading to cell death (apoptosis) due to an inability of DNA to replicate. (Ang et al., 2005) However, Cis, like many other chemotherapeutic drugs, is passive (*i*.*e*., non-targeting), non-selective, and has short circulation in the blood–properties that can cause insufficient therapeutic dosage leading to low efficacy, drug resistance, and therapeutic failure. (Coates et al., 1983; Lage, 2008)

Targeted drug delivery systems have been championed as the next generation of active chemotherapy treatments as they are capable of actively targeting tumor cells for selective release of their chemotherapeutic drug cargo. (Pinzani et al., 1994; Xiao et al., 2012) These properties minimize side effects to healthy cells, reduce systemic toxicity, and enhance the efficiency of the chemotherapeutic drug. (Yuan et al., 2014) Furthermore, these systems provide protection against degradation of the chemotherapeutic drugs and ensure proper dosage to the targeted cells. (Allard et al., 2009) Of the many strategies for developing targeted drug delivery systems, the employment of tumor homing peptides stand out as particularly advantageous given that these peptides specifically bind to receptors that are overexpressed on the surface of cancer cells. (Worm et al., 2020) For example, the short peptide motif, Arg-Gly-Asp (RGD), is specifically recognized by several αV integrin receptors that are frequently overexpressed in cancer cells. (Nieberler et al., 2017) The general mechanism of RGD’s activity is akin to that of a ‘homing device’, in which the RGD-functionalized nanosystem navigates to a cancer cell, docks as a result of the selective interactions with specific integrin receptors, and delivers the chemotherapeutic agent. (Temming et al., 2005; Katsamakas et al., 2017) Several examples of using the RGD peptide motif to direct nano-based drug delivery systems to cells have been reported with varying degrees of success and therapeutic efficacy and efficiency. (Spicer et al., 2018)

Zeolitic imidazolate frameworks (ZIFs) are metal-organic analogues of inorganic zeolites. (Phan et al., 2010; Zha et al., 2022) Nano-sized ZIFs (nZIFs) are useful delivery systems due to their biocompatibility, adjustable pore sizes, large surface area (*i*.*e*., high drug storage capacity), aqueous stability, pH-responsive behavior, and, most importantly, their ability to be functionalized pre- or post-synthetically. (Nguyen et al., 2014; Nguyen et al., 2016; Abdul Kamal et al., 2022) Indeed, nZIFs have widely been used as delivery systems for 5-fluorouracil, doxorubicin and quercetin, among others, in conventional cancer chemotherapy. (Jiang et al., 2018; de Moura Ferraz et al., 2020; Ho et al., 2020; Tan et al., 2021) One nZIF system, nZIF-90, is attractive in particular due to its biocompatibility with different cell lines, chemical stability, and its ability to be post-synthetically modified through a free carboxaldehyde functional group that is attached to the imidazolate linker backbone. (Morris et al., 2008) Though these features are widely known, covalent post-synthetic modification of nZIF-90 with a specific cancer-targeting ligand, to create an active, targeting drug delivery system for treating cancer cells with enhanced specificity and efficacy has seldom been reported in cancer treatment. (Horcajada et al., 2010)

**Scheme 1:**
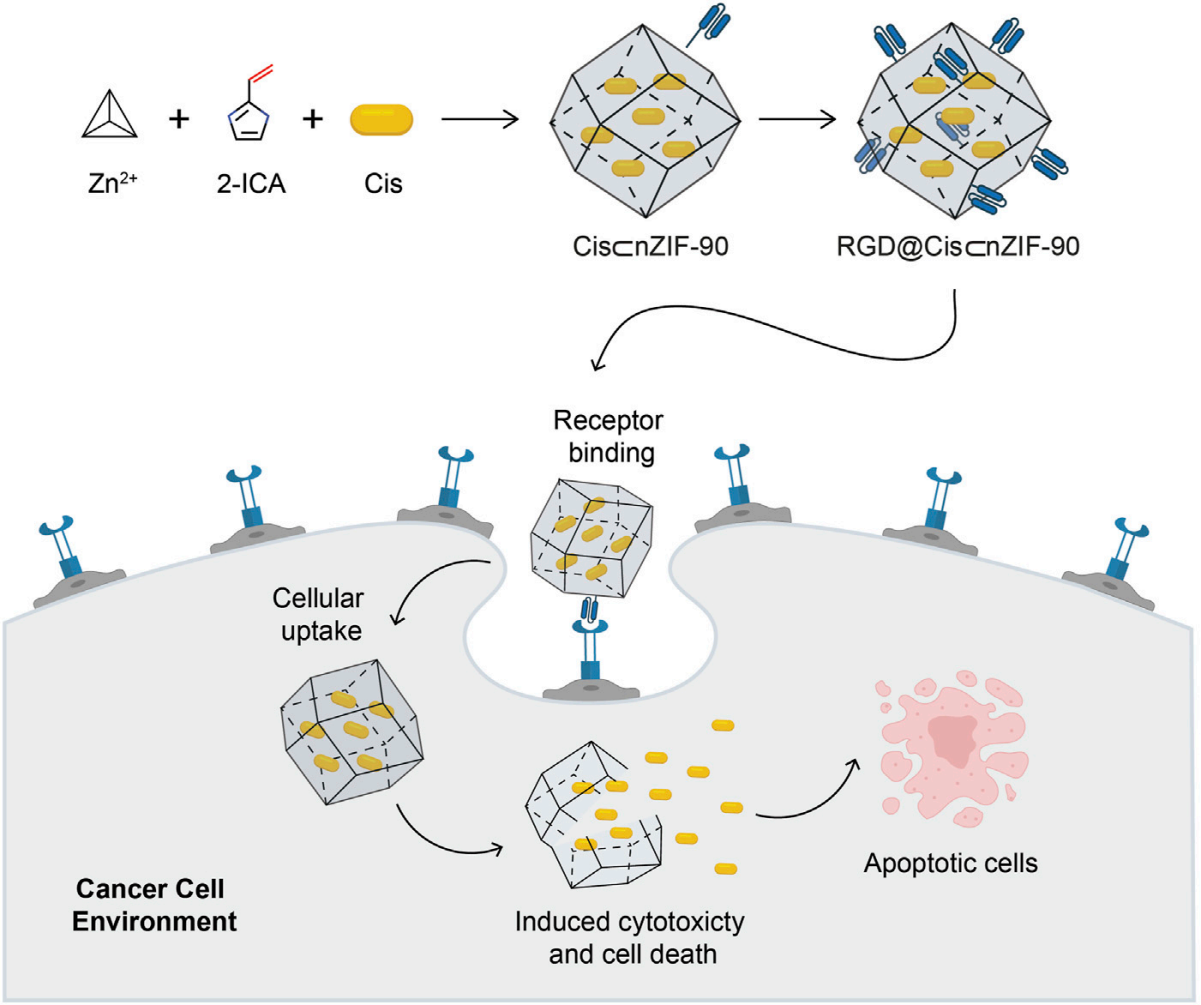
Schematic illustration for the synthesis of the Cis-encapsulated nZIF-90 modified with RGD, RGD@Cis⊂nZIF-90, and its ability for targeted cancer treatment. The active delivery nanosystem is capable of cellular uptake and inducing cytotoxicity, cell death, and apoptosis. Atom colours for 2-ICA: C, black; N, blue; O, red.

In this study, we report the synthesis, physicochemical properties, and biocompatibility of nZIF-90, as a nanocarrier loaded with Cis and covalently functionalized with RGD homing peptide, to actively target and selectively deliver Cis to lung cancer cells (Scheme 1). When compared to other nZIF delivery systems, nZIF-90 possesses free carboxaldehyde functional groups that are exploited for covalent functionalization with the amino group of the RGD homing peptide motif. (Morris et al., 2008) This ensures the attachment of the RGD homing peptide on the surface of nZIF-90 to make it an active delivery system. Although the parent, unfunctionalized nZIF-90 lacks active targeting properties itself, previous research has demonstrated that its surface can be modified *via* a variety of reaction pathways and therapeutic agents can be efficiently loaded within and released from its pores. (Zhang et al., 2017) Our report, detailed herein, expands upon these previous studies by demonstrating that Cis-encapsulated nZIF-90 modified with RGD has favorable biocompatibility and pH-responsive properties, thereby making it a viable candidate for targeted tumor therapy. Following the successful realization of this nano-delivery system, we further demonstrate its efficacy and efficiency in actively transporting and delivering Cis to cancer cells.

## Materials and methods

### Materials and supplies

Imidazole-2-carboxaldehyde (2-ICA; 99% purity), zinc nitrate hexahydrate (Zn(NO_3_)_2_∙6H_2_O; 99% purity), polyvinylpyrrolidone (PVP; average mol. wt. = 40000), *p*-formaldehyde (95% purity), phosphate buffered saline (PBS), fluorescein (95% purity), arginine-glycine-aspartic acid (RGD), cisplatin (Cis; 99% purity), hydrochloric acids, and dimethyl sulfoxide (DMSO; 99.5% purity) were obtained from Sigma Aldrich. Methanol (99.9% purity) and tert-butyl alcohol (*t*-BuOH; 99% purity) were purchased from Fisher Scientific. RMPI 1640, fetal bovine serum (FBS), 3-(4,5-dimethylthiazol-2-yl)-2,5-diphenyltetrazolium bromide (MTT), and trypsin were acquired from Gibco (Grand Island, USA). Hoechst33342 was procured from Invitrogen. Annexin V-FITC/propidium iodide (PI) apoptosis detection kit was received from Elabscience. A Milli-Q gradient water system was used to produce ultra-pure water. Normal human lung fibroblast cells (MRC-5 cells) and adenocarcinoma human alveolar epithelial cells (A549 cells) were obtained from the American Type Culture Collection (ATCC). All materials detailed herein were used without further purification.

### Material characterization and cellular analysis techniques

Fourier transform infrared (FT–IR) spectra were recorded over a frequency of 4,000–400 cm^−1^ using a Nicolet 6700 FT–IR attenuated total reflection (FT–IR–ATR) spectrophotometer. Powder X-ray diffraction (PXRD) analyses were carried out on a PANalytical X'Pert Pro-MPD diffractometer equipped with an image plate detector in continuous scan mode with Cu Kα (λ = 1.54178 Å). For each PXRD measurement, samples were prepared at room temperature (25°C) by transferring dried powder to a sample holder, which was flattened using a spatula. Ultraviolet–visible (UV-Vis) spectroscopy measurements were recorded at wavelengths ranging from 200 to 400 nm using a Shimadzu UV-2600 UV–Vis spectrophotometer. Energy dispersive spectroscopy analysis of X-ray (EDX) to show the presence of all elements in nZIF-90 and Cis⊂nZIF-90. Size exclusion chromatography (SEC) was performed on an Akta Avant system using a Superdex peptide column 10/300 (GE Healthcare). Solid-state cross-polarization magic angle spinning nuclear magnetic resonance spectroscopy (^13^C CP–MAS NMR; 100.62 MHz) was measured using a Bruker Ascend 400 MHz NMR with a 4 mm zirconia rotor size and spinning rate of 12 kHz. Field-emission scanning electron microscopy (FE–SEM) analysis was carried out using a JEOL JSM-7600F instrument to determine the particle size and sample morphology. A Zetasizer Nano ZS Malvern dynamic light scattering (DLS) instrument was employed to determine the surface charge and hydrodynamic size of all nanoparticles in this study. All samples were diluted eight-fold in methanol relative to their initial concentration prior to performing the DLS experiments. Thermogravimetric analysis (TGA) was performed on a PerkinElmer STA6000 thermal analysis system with the sample held in an alumina pan heated from 50 to 800°C at a rate of 10°C min^−1^ under continuous N_2_ flow. A Carl Zeiss fluorescence microscope was used to image cellular uptake. The images were captured using a Zeiss AxioCam MRm camera and analysed using Zen Lite 2012 software. The absorbance of formazan crystals was determined at 570 nm using a Biotek ELx800 universal microplate reader. In the final analysis, apoptotic cell population was quantified using a BD LSRFortessa flow cytometer with FlowJo_V10.7.2 being employed to analyze the corresponding plots.

### Synthesis and pH stability of nano-ZIF-90 (nZIF-90)

Nano-sized ZIF-90 was synthesized by adopting a previously described method, but with minor modifications. (Shieh et al., 2013) In the synthesis reaction, Zn(NO_3_)_2_∙6H_2_O (297 mg, 0.996 mmol) was dissolved in 16 ml of *t*-BuOH with stirring. In a separate vial, 2-ICA (384 mg, 4.00 mmol) and PVP (10 mg) were dissolved in 16 ml of deionized water at 45°C while sonicating until completely dissolved. After 5 min of sonication, the two solutions were mixed. Formation of precipitate was observed and after 5 min the mixture was centrifuged for 10 min at a speed of 10,000 revolutions per min (rpm). The obtained solid was collected *via* filtration, washed with methanol (6 × 3 ml), and vacuum dried at room temperature for 12 h to yield as-synthesized ZIF-90 as a white crystalline solid.

### Synthesis of cisplatin-encapsulated nZIF-90 (Cis⊂nZIF-90) and quantification of loading amount

A previous method was modified to obtain a one-pot method of producing Cisplatin encapsulated within nZIF-90 (Cis⊂nZIF-90). (Eaton et al., 2017) Specifically, Zn(NO_3_)_2_∙6H_2_O (297 mg, 0.996 mmol) and Cis (8 mg, 0.027 mmol) were dissolved in a *t*-BuOH/H_2_O (1:1, 32 ml) solution. This solution was then poured into a vial containing a solution of 2-ICA (384 mg, 3.996 mmol) and PVP (10 mg) in water (48 ml). The mixture was sonicated for 5 min at room temperature. The mixture was then centrifuged (10000 rpm, 10 min), washed with excess methanol, and vacuum dried at room temperature for 12 h to obtain the as-synthesized Cis⊂nZIF-90. Quantification of Cis encapsulated within nZIF-90 was determined by UV–Vis spectroscopy. To release the Cis encapsulated within nZIF-80, Cis⊂nZIF-90 (5 mg) samples were precisely weighed in triplicate and placed in PBS solutions (pH = 5) for 24 h. This resulted in the complete dissociation of Cis⊂nZIF-90 and 100% release of Cis. After 24 h, the samples were centrifuged and the Cis concentration in the supernatant was determined using the λ_max_ of Cis (302 nm) *via* UV–Vis. The concentration in solution was calculated using a pre-established calibration curve with the results being presented as mean ± standard deviation (SD) of three UV-Vis measurements. As a control experiment, parent nZIF-90 samples were degraded using the same conditions and their supernatant was used to detect the presence of a background signal. The following equations were employed to calculate the percentage of Cis loading (CL) within nZIF-90: CL% = [(initial weight (mg) of Cis incorporated within nZIF-90)–(weight (mg) of Cis unincorporated)]/[(initial weight (mg) of Cis incorporated within nZIF-90)] × 100.

### Post-synthetic modification of Cis⊂nZIF-90 with RGD homing peptide (Cis⊂RGD@nZIF-90)

Following previous reports, the RGD homing peptide (2.0 mg, 0.0056 mmol) was first dissolved in MeOH (10 ml). (Blanco et al., 2015) Next, Cis⊂nZIF-90 (20 mg) was added to the RGD-containing solution and the resulting mixture was stirred for 48 h at room temperature. After 48 h, the solid was collected by centrifugation at 10,000 rpm for 8 min and washed with excess MeOH before being immersed in MeOH for 24 h to remove unreacted RGD. The solid was then vacuum dried for 12 h at room temperature. To determine the chemical stability of RGD@Cis⊂nZIF-90, The sample (50 mg) was dispersed in 50 ml of simulated lung fluid (SLF) solutions with pH values of 7.4 and 6.0, respectively. The resulting dispersions were incubated in an incubator shaker for 24 h at 37°C and 100 rpm. Following the incubation, the samples were centrifuged, washed with methanol, vacuum dried at room temperature, and analysed using PXRD. Size exclusion chromatography (SEC) was used to determine the degree of post-synthetic modification of Cis⊂nZIF-90. For this measurement, RGD@Cis⊂nZIF-90 (50 µl) was digested using a hydrochloric acid solution (2 M, 50 µl). At room temperature, the digested solution was evaluated by SEC using a 20% MeOH to H_2_O ratio at a flow rate of 0.5 ml min^−1^. The presence of RGD was confirmed by comparing the peaks of digested RGD@Cis⊂nZIF-90 to that of pure RGD.

### Synthesis of fluorescein (FI)-Encapsulated nZIF-90 (FI⊂nZIF-90) and RGD@nZIF-90 (RGD@FI⊂nZIF-90)

Fluorescein (FI) was encapsulated within nZIF-90 and RGD@ZIF-90 following the same procedure as described for Cis with the exception that Cis was substituted for FI in the recipe to afford FI⊂nZIF-90 and RGD@FI⊂nZIF-90, respectively. (Mei et al., 2015)

### *In vitro* drug release and cellular uptake studies

A dynamic dialysis technique was used to assess *in vitro* drug release. In a typical measurement, a solution containing RGD@Cis⊂nZIF-90 (1 ml, 10 mg ml^−1^) was prepared in PBS and introduced to a dialysis bag, which, in turn, was dialyzed in PBS (49 ml) at pH = 7.4 and 5.0 in an incubator shaker at 37°C and 100 rpm for 24 h. At predetermined time intervals, 1 ml of sample was taken from the released medium, and the same volume of media was reintroduced to the released medium. The samples were collected in triplicate and were immediately analysed by UV–Vis spectroscopy to determine the cumulative drug release. For cellular uptake analysis, MRC-5 and A549 cell lines were cultured in RPMI-1640 containing 10% fetal bovine serum (FBS) and 1% antibiotic penicillin-streptomycin solution. All cell lines were incubated at 37°C under a humidified atmosphere that contained 5% CO_2_. Right after confluence, the cells were sub-cultured into a new culture media and cellular studies were then conducted. To visualize cellular uptake, a fluorescence microscope was employed when using FI⊂nZIF-90 and RGD@Cis⊂nZIF-90 samples. A549 cells were seeded at a density of 3 × 10^5^ in 6-well culture plates and cultured for 24 h at 37°C in a 5% CO_2_ humidified atmosphere using RPMI-1640 containing 10% fetal bovine serum with pH = 7.4. These cells were then treated for 3 h with FI⊂nZIF-90 and RGD@FI⊂nZIF-90 at equivalent concentrations (12 μg ml^−1^). Following treatment, the cells were washed with PBS and fixed with *p*-formaldehyde three times. Untreated cells were used as controls. The cells were then stained with Hoechst 33342 for 20 min and then washed three times with PBS.

### Cellular cytotoxicity assays

MTT assays were used to determine the cytotoxicity of Cis, nZIF-90, Cis⊂nZIF-90, and RGD@Cis⊂nZIF-90. MRC-5 and A549 cells were seeded on a 96-well plate with a cell density of 3 ×10^3^ cells well^−1^ and allowed to attach for 24 h, respectively. After 24 h, the cells were treated with Cis, nZIF-90, Cis⊂nZIF-90, and RGD@Cis⊂nZIF-90 for 24 and 48 h. The concentration ranged from 0 to 100 µg ml^−1^. Following treatment, each well was filled with 20 µl of MTT solution (5 mg ml^−1^ in PBS) and incubated for 3 h. Each well was then diluted with 80 µl DMSO to solubilize the formazan crystals. The absorbance (Abs) at 570 nm was determined using a Biotek ELx800 universal microplate reader. The following formula was used to determine the percentage of viable cells:

$$Cell\;viability\;\left(\%\right)={Abs}_{treated\;Cells}∕{Abs}_{Untreated\;Cells}\times 100$$

(Zheng et al., 2018)

The 50% inhibition of cell growth (IC_50_) values for both MRC-5 and A549 cells were calculated using curve-fitting methods and statistical analysis software. The selectivity index (SI) towards cancer cells for Cis, nZIF-90, Cis⊂nZIF-90, and RGD@Cis⊂nZIF-90 were calculated using the IC_50_ values for both MRC-5 and A549 cells as follows:

$$SI={IC}_{50\left(MRC-5\right)}/{IC}_{50\left(A549\right)}$$

(Braga et al., 2020)

### Cellular apoptosis and statistical analyses

The ability of various samples to induce apoptosis was investigated by determining the percentage of late and early apoptotic cells using Annexin V-FITC and PI dual staining. MRC-5 and A549 cells were seeded at a density of 3 × 10^5^ cells well^−1^ in 6-well plates and incubated at 37°C for 24 h. After 24 h, the media was removed, and the cells were washed with PBS. The cells were then treated for an additional 48 h with Cis, nZIF-90, Cis⊂nZIF-90, and RGD@Cis⊂nZIF-90 at concentrations equivalent to the IC_50_ value. No treatments were added to control wells. After 48 h of incubation, the adhered cells were trypsinized with trypsin and washed with PBS, followed by the addition of 100 µl of 1× binding buffer. Annexin V-FITC (2.5 µL) and PI (2.5 µl) were added to the 1× binding buffer-containing cells and the cell mixtures were incubated for 15 min at 25°C. Following this period, 1× binding buffer (400 µl) was added prior to performing flow cytometric analysis. A minimum number of 10,000 cells were counted for each sample. Apoptosis was computed as the sum of Q_2_ + Q_3_. (Zeng et al., 2012) Graph Pad Prism 8.0 was used to conduct all statistical analyses as depicted in these experiments. The data were expressed as mean ± SD from at least triplicate observations. A statistically significant difference was defined as one with a *p*-value < 0.05.

## Results and discussion

### Synthesis, characterization, drug loading and encapsulation efficiency

ZIF-90 was chosen as the carrier of cisplatin (Cis) for primarily two reasons: 1) ZIF-90 has an abundance of carboxaldehyde functional groups from its building block imidazolate linker accessibly situated on its surface, which provides an opportunity for post-synthetic modification with the RGD homing peptide. This simple structural feature enables ZIF-90 to be transformed into an active carrier for an important chemotherapeutic agent; and 2) nanocrystals of ZIF-90 can easily be obtained in the sub-200 nm size regime using straightforward green conditions, which ensures that the resulting drug delivery nanosystem can achieve maximal cellular uptake. Accordingly, the synthesis of nano-sized ZIF-90 (nZIF-90) and Cis-encapsulated nZIF-90 (Cis⊂nZIF-90) was performed *via* a one-pot reaction of Zn(NO_3_)_2_∙6H_2_O, imidazole-2-carboxaldehyde (2-ICA), polyvinylpyrrolidone, and Cis in *t*-BuOH and H_2_O at 45°C. Post-synthetic modification of Cis⊂nZIF-90 with the RGD homing peptide was then carried out by simply immersing Cis⊂nZIF-90 in a methanolic solution of RGD for 48 h at room temperature to yield RGD@Cis⊂nZIF-90. Prior to structural characterization, nZIF-90, Cis⊂nZIF-90, and RGD@Cis⊂nZIF-90 were washed with methanol to remove unreacted starting materials and/or by-products, and then solvent-exchanged and activated.

**FIGURE 1:**
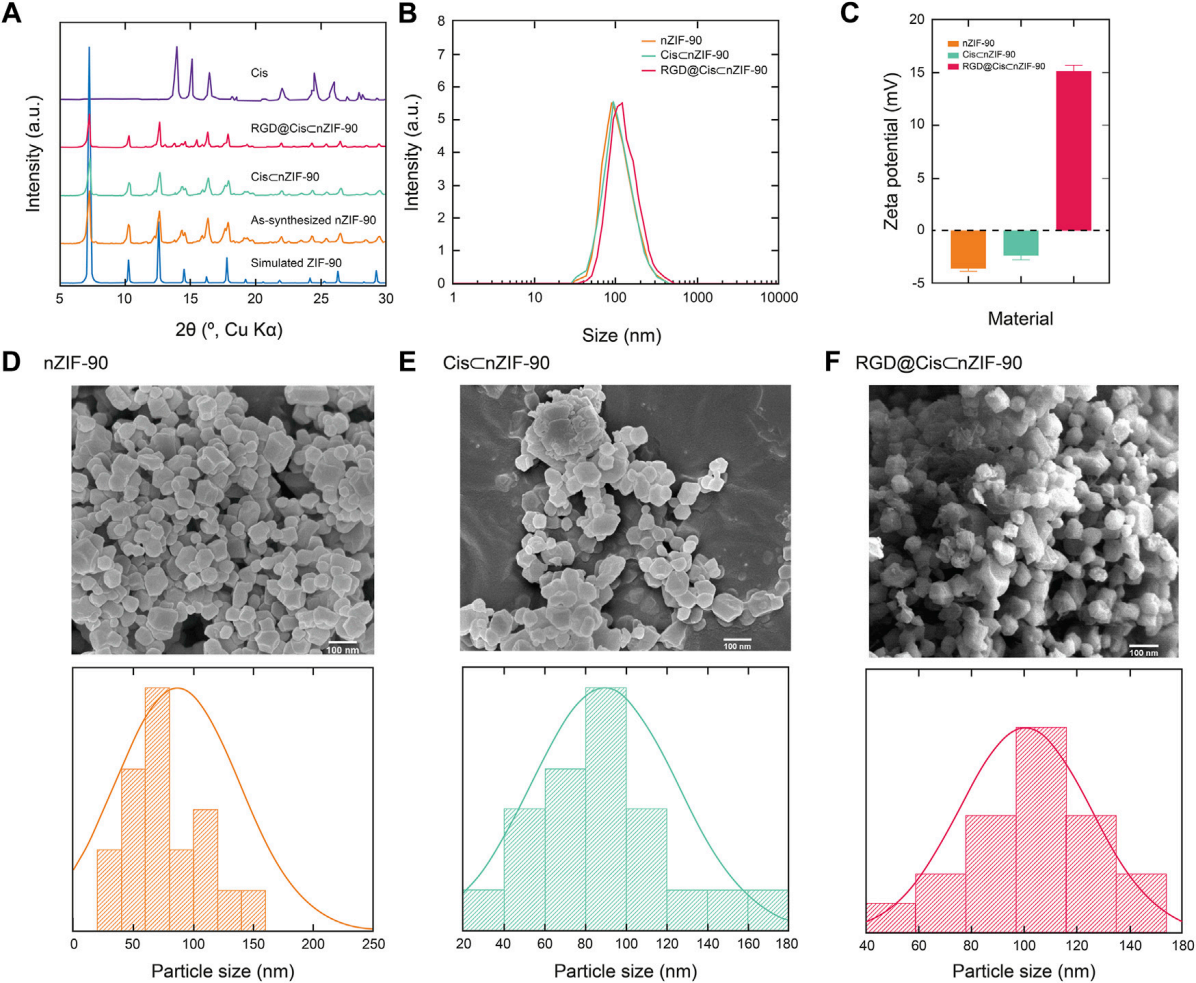
**(A)** Powder X-ray diffraction (PXRD) patterns for the simulated nZIF-90 crystal structure (blue), as-synthesized nZIF-90 (orange), Cis⊂nZIF-90 (green), RGD@Cis⊂nZIF-90 (red) and Cis (purple). **(B)** Dynamic light scattering particle size distribution of nZIF-90, Cis⊂nZIF-90, and RGD@Cis⊂nZIF-90. **(C)** Zeta-potential of nZIF-90, Cis⊂nZIF-90, and RGD@Cis⊂nZIF-90. Field-emission scanning electron microscopy (FE-SEM) images with the corresponding size distribution histograms of **(D)** nZIF-90 **(E)** Cis⊂nZIF-90, **(F)** RGD@Cis⊂nZIF-90.

The crystallinity and phase purity of nZIF-90 were assessed *via* powder X-ray diffraction (PXRD) analysis. The diffraction pattern for nZIF-90 exhibited strong, sharp peaks centered at 2θ = 7.27, 10.26, 12.62, 14.48, 16.20, and 17.78°, which correspond to the (110), (200), (211), (220), (310), and (222) Miller indices, respectively. The diffraction peak positions in the patterns of Cis⊂nZIF-90, and RGD@Cis⊂nZIF-90 were found to be coincident to those of nZIF-90 and, more importantly, to the diffraction pattern simulated from the single crystal X-ray data (Figure 1). Taken together, all synthesized samples were observed to be highly crystalline and phase pure. To ensure that the crystals were within the nanosize regime, a combination of field-emission scanning electron microscopy (FE–SEM) and dynamic light scattering (DLS) measurements were undertaken. In the FE–SEM images (Figure 1), crystallites with rhombic dodecahedron morphology were observed with nanoscale size (size distributions centered at 86, 94, and 104 nm for nZIF-90, Cis⊂nZIF-90, and RGD@Cis⊂nZIF-90, respectively). This is an important observation for this study as nanoparticles are required for uptake and cytotoxicity within cancer cells. (Foroozandeh and Aziz, 2018) It is noted that the presence of RGD on the surface of nZIF-90 led to an increase in the size of the aggregated nanoparticles. In comparison to the FE–SEM results, DLS measurements produced similar average hydrodynamic sizes of 93, 96, and 116 nm for nZIF-90, Cis⊂nZIF-90, and RGD@Cis⊂nZIF-90, respectively. Hydrodynamic size, as determined by DLS (Figure 1), typically results in larger values compared with those obtained by FE–SEM analysis as the hydration layer surrounding the nanoparticles is included within the measured size. (Yang et al., 2019) Finally, zeta potential analyses (Figure 1) were employed to determine the surface charge density of the dispersed nanoparticles. The measured zeta potential values were –3.6, –2.3, and +15.2 mV for nZIF-90, Cis⊂nZIF-90, and RGD@Cis⊂nZIF-90, respectively, with the change in surface charge moving from negative to positive because of electron-donating groups (-NH_2_) on the surface of RGD@Cis⊂nZIF-90 that are not present in nZIF-90 nor Cis⊂nZIF-90. The positive zeta potential value for RGD@Cis⊂nZIF-90 is indeed promising as it is known that positive surface charges increase cell surface affinity and uptake of nanoparticles in tumor cells. (Suarasan et al., 2016)

**FIGURE 2:**
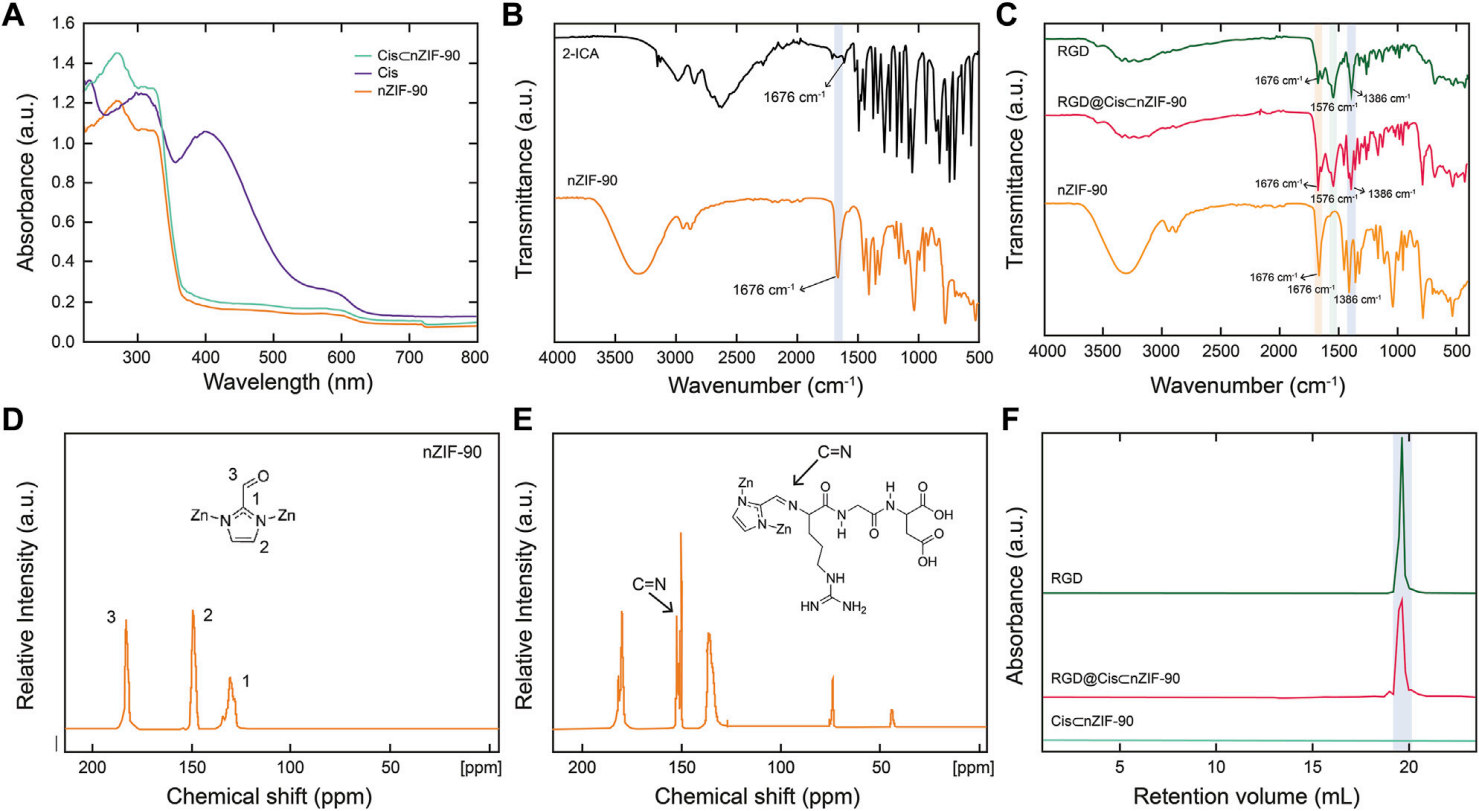
**(A)** Solid-state UV-vis of nZIF-90 (orange), Cis (purple) and Cis⊂nZIF-90 (green). **(B)** FT-IR spectra of 2-ICA linker (black) compared with nZIF-90. **(C)** FT-IR spectra comparison of nZIF-90, RGD@Cis⊂nZIF-90 (red) and RGD (dark green). **(D,E)** Solid state ^13^C CP-MAS NMR spectra of nZIF-90 **(D)** and RGD@ZIF-90 **(E)**. **(F)** Size exclusion chromatograms comparing acid-digested Cis⊂nZIF-90 and RGD@Cis⊂nZIF-90 with the chromatogram of free RGD-peptide.

With the crystallinity, phase purity, and nano-size established, attention turned to quantifying encapsulation of Cis within the pores of nZIF-90. The encapsulation process was ultimately achieved using a one-pot pre-synthetic procedure with attempts to load Cis post-synthetically being unsuccessful. Solid-state UV–Vis spectroscopy (Figure 2) was first used to quantify encapsulation of Cis, however, no UV absorption band for Cis in Cis⊂nZIF-90 was observed indicating suppression by nZIF-90 and/or the presence of a self-quenching effect. In order to release and, hence quantify, the Cis encapsulated within nZIF-90, Cis⊂nZIF-90 was digested to promote complete dissociation of the framework and release of the chemotherapeutic agent. The concentration of Cis in the supernatant was then determined using the λ_max_ of Cis (302 nm) *via* solution-state UV–Vis spectroscopy. The loading efficiency in Cis⊂nZIF-90 was found to be high at 24.83 ± 0.8 wt%. To provide further confirmation of the loading efficiency, inductively coupled plasma–optical emission spectroscopy was performed to quantify the amount of Pt within the Cis⊂nZIF-90 system. Accordingly, a Pt loading of 0.46 wt% was found, which is equivalent to a Cis loading efficiency of 24 wt%; a value that matches the one calculated from the solution-state UV-Vis measurements. Additionally, energy dispersive X-ray analysis (EDX) for the loading of cisplatin showed the existence of carbon, zinc, nitrogen, oxygen, and platinum elements (Supplementary Figures S1, S2) and confirmed the weight percentages (wt%) of C, Zn, N, O, and Pt respectively as 45.0 ± 0.6, 33.7 ± 0.5, 12.1 ± 0.6, 7.7 ± 0.3, and 1.5 ± 0.3 wt%.

### Characterization of the covalent functionalization of nanocarrier with RGD peptide

Post-synthetic modification of Cis⊂nZIF-90 with RGD homing peptide was characterized by Fourier transform infrared spectroscopy (FT–IR), solid state ^13^C cross polarization magic angle spinning nuclear magnetic resonance spectroscopy (^13^C CP–MAS NMR), and size exclusion chromatography (SEC). The RGD was covalently attached to the surface of Cis⊂nZIF-90 by the formation of a C=N bond for RGD@Cis⊂nZIF-90. When the FT-IR spectra of RGD, nZIF-90, and RGD@Cis⊂nZIF-90 were compared, it was discovered that RGD@Cis⊂nZIF-90 had more absorption bands than nZIF-90, which can be attributed to functional groups in the RGD modified section. The stretching absorption band (C=O) for the aldehyde functional group attached on the 2-ICA linker is at 1,675 cm^−1^, and the C–N absorption band for the 2-ICA linker is at 1,386 cm^−1^ (Figure 2B). The RGD@CisnZIF-90 FT–IR spectrum revealed a new C=N absorption band (1,576 cm^−1^) indicating imine formation and, thus, successful RGD binding on the surface of Cis⊂nZIF-90 (Figure 2C). Covalent bond formation between Cis⊂nZIF-90 and the RGD homing peptide was proven *via* ^13^C CP–MAS NMR. In the spectra for both the parent nZIF-90 and RGD@Cis⊂nZIF-90 (Figure 2D), expected resonances at 130, 150, and 178 ppm were observed, which represent the symmetrically equivalent C_2_, C_1_, and C_3_ carbons of 2-ICA linker, respectively. Importantly, the presence of an imine carbon at 154 ppm was found only in the spectrum of RGD@Cis⊂nZIF-90 (Figure 2E) indicative of a successful imine condensation reaction having taken place between the aldehyde functional groups of the 2-ICA linker and the terminal amine group of the RGD homing peptide. Finally, SEC of digested pristine RGD, Cis⊂nZIF-90, and RGD@Cis⊂nZIF-90 (Figure 2F) were performed to provide final confirmation of successful post-synthetic modification. As expected, there was no RGD peak observed in the chromatograms of Cis⊂nZIF-90 whereas RGD peaks were found at the same retention volume in the chromatograms of both pure RGD and RGD@Cis⊂nZIF-90.

### *In vitro* pH responsive drug delivery

**FIGURE 3:**
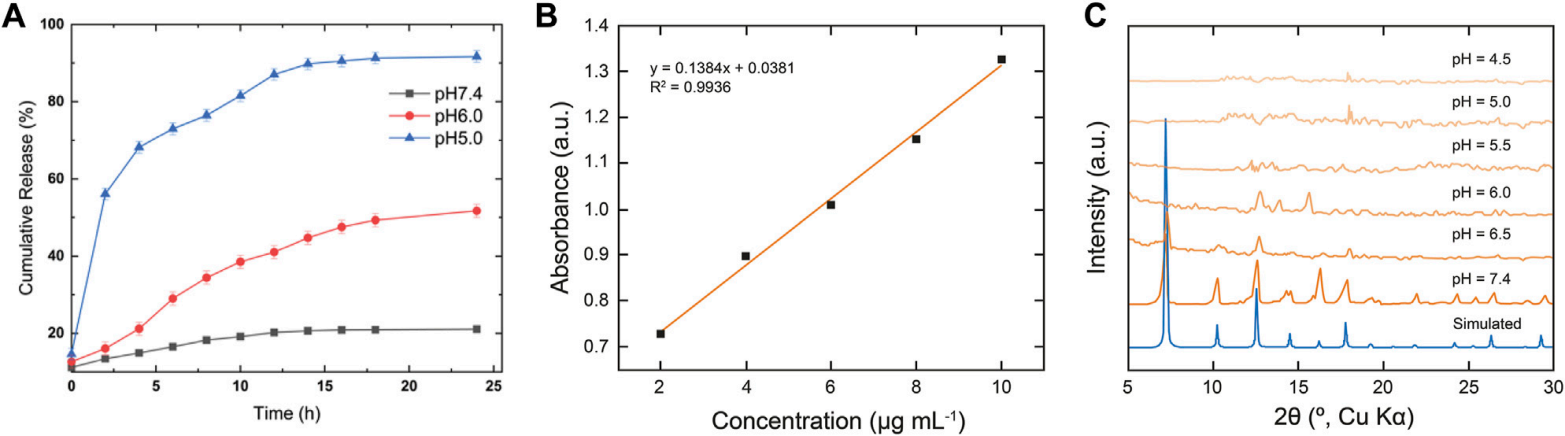
**(A)** *In vitro* cumulative release of Cis from RGD@Cis⊂nZIF-90 in PBS at pH = 7.4 (black squares), 6.0 (red circles), and 5.0 (blue triangles) for 24 h at 37°C **(B)** Calibration curve of Cis at 302 nm. **(C)** PXRD data comparing the simulated pattern of nZIF-90 to experimental nZIF-90 samples immersed in different pH solutions.

After successful structural confirmation of the RGD@Cis⊂nZIF-90 nano-delivery system, we sought to examine its drug release behavior in different pH environments. Accordingly, the pH-responsive behavior of RGD@Cis⊂nZIF-90 was evaluated by monitoring the release profile of Cis from the nano-delivery system in a PBS solution at pH = 5.0, 6.0, and 7.4 at 37°C (Figures 3A,B). It is noted that pristine Cis was dialyzed as a control system. At pH = 5.0, a rapid Cis release of 50.056 ± 0.82% was observed less than the first 2 h leading to a maximum Cis release of 91.73 ± 1.51% obtained after 9 h. For the measurements at pH = 6.0 and 7.4, only 51.73 ± 1.71% and 21.08 ± 0.13% release of Cis was observed even after 24 h. As expected, there was more Cis release within an acidic pH–a satisfactory result given that this environment is characteristic of a tumoral cell–due to framework dissociation and Cis release. The framework dissociation was shown in Figure 3C *via* PXRD analysis of nZIF-90 after immersing it in PBS solutions whose pH = 6.0 or lower for 24 h. Specifically, in these diffraction patterns the peak at 2θ = 7° disappeared, thereby suggesting framework dissociation. It is noted that when nZIF-90 was immersed in PBS solutions higher than pH = 6.0, the diffraction patterns revealed that the framework had maintained its crystalline structure and stability under physiological conditions. The pH-responsive behaviour of the nZIF-90-based delivery nanosystems is important for limiting the release of Cis to physiologically relevant pH environments. We also investigated the stability of RGD@Cis-nZIF-90 in simulated lung fluid (SLF) at pH = 7.4 and 6.0, which provides a realistic representation of a lung cancer environment. These studies were conducted using the same technique. It was discovered that the RGD@CisnZIF-90 particles’ crystalline structure and stability in SLF at pH = 7.4 remained stable after 24 h of incubation. The crystalline structure of the RGD@CisnZIF-90 particles decreased in SLF at pH = 6.0 (Fig. S3) owing to the RGD@CisnZIF-90 being more responsive in an acidic environment than in a neutral pH.

### *In vitro* cell analysis

**FIGURE 4:**
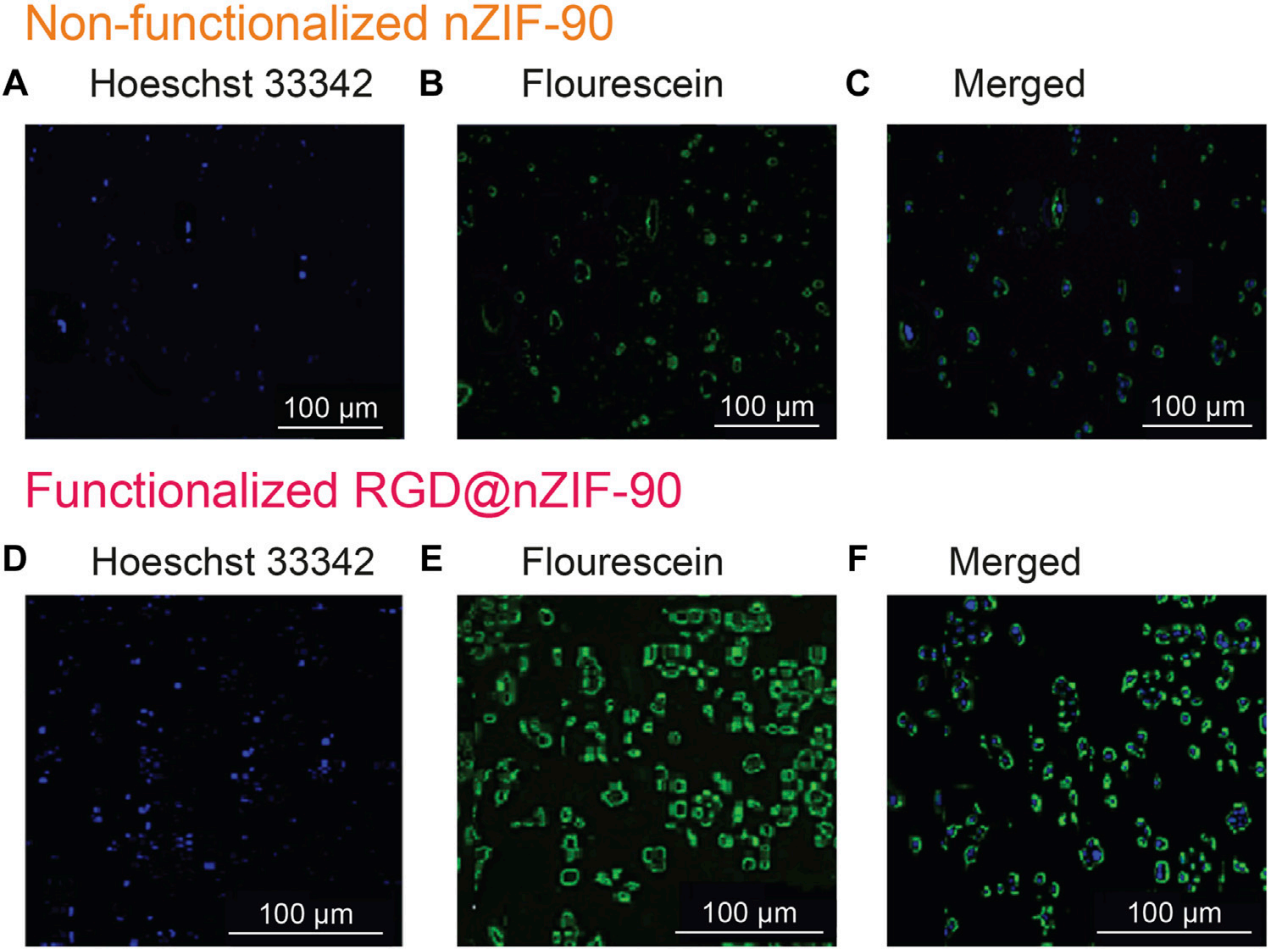
Differential cellular uptake of nanoparticles in A549 cells with fluorescence microscopy images showing nanoparticles localized in different cellular compartments: for the non-fictionalized FI⊂nZIF-90, **(A)** Hoechst 3342, **(B)** Fluorescein, **(C)** merged images and for the RGD-functionalized RGD@FI⊂nZIF-90, **(D)** Hoechst 3342, **(E)** Fluorescein, and **(F)** merged images.

**FIGURE 5:**
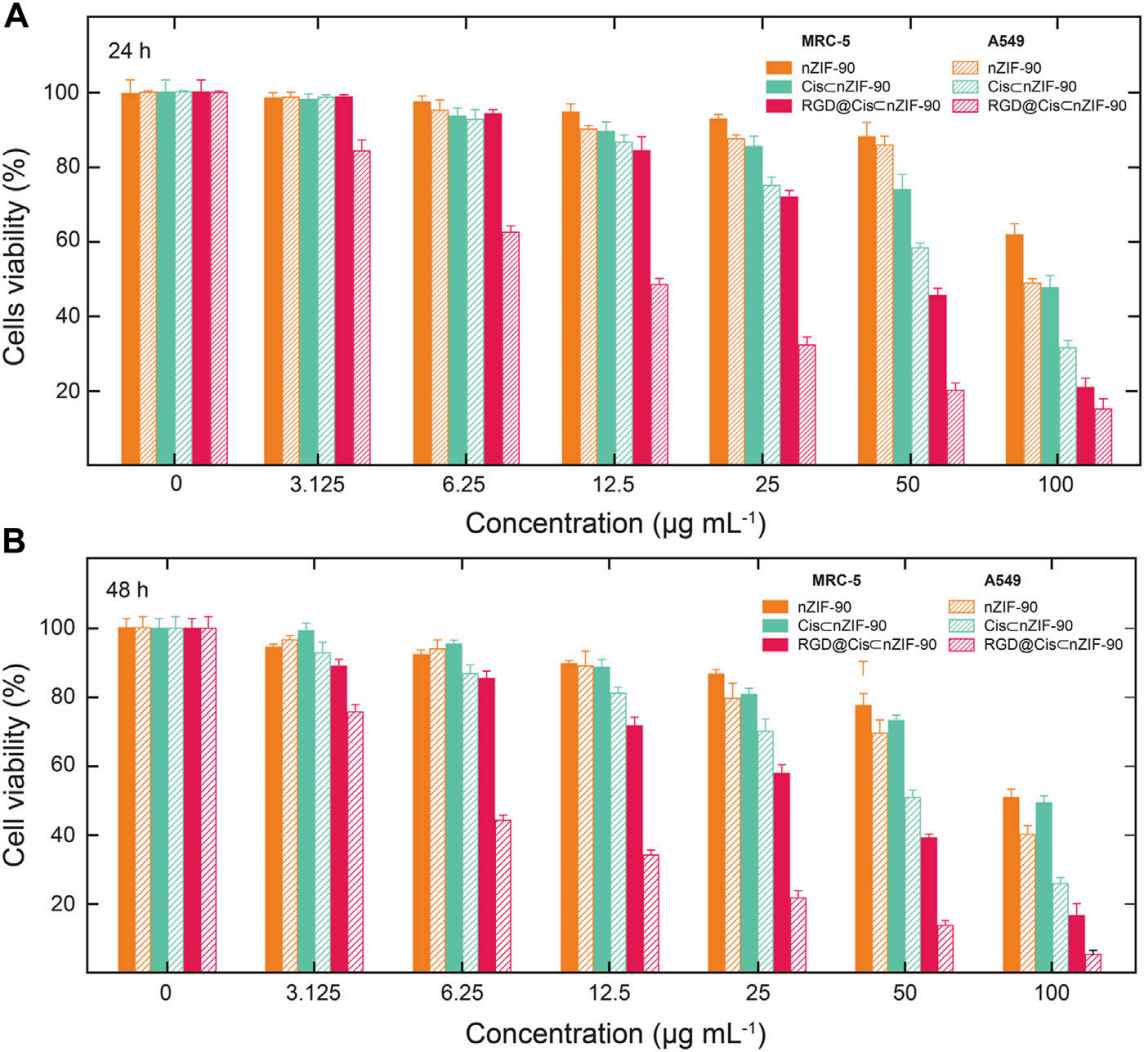
Cytotoxicity was assessed by MTT assay following treatment of A549 cells and MRC-5 cells with nZIF-90 (orange), Cis⊂nZIF-90 (green) and RGD@Cis⊂nZIF-90 (red) for **(A)** 24 h and **(B)** 48 h. Percentages of cell viability are presented as a function of nanoparticle concentration used.

Quantification of the cellular uptake is critical for measuring the effectiveness of a nanoparticle as a drug delivery nanosystem. For this, Cis was replaced with a fluorescence label, fluorescein (FI), to visualize the *in vitro* cellular uptake of FI⊂nZIF-90 and RGD@FI⊂nZIF-90 within an adenocarcinoma alveolar epithelial (A549) cell line after 3 h of treatment (Figure 4). Visualization of the uptake was monitored using a fluorescence microscope by following the counter staining of cell nuclei using Hoechst33342 (blue fluorescence). Significantly stronger fluorescence intensity was observed in A549 cells after treatment with RGD@FI⊂nZIF-90 than FI⊂nZIF-90. This result suggests that there was a higher degree of accumulation and aggregation within A549 cells when the delivery nanosystems was functionalized with the RGD homing peptide. As is shown in the merged images of Figure 4F, FI is released from the nZIF-90 nano-delivery system and diffuses throughout the cell. This study confirms the RGD@FI⊂nZIF-90 system’s ability to bind with specific integrin receptors (α_V_β_3_) present on the surface of the A549 cell membranes, thereby displaying enhanced translocation and endothelialisation. Furthermore, these results indicate that the RGD@FI⊂nZIF-90 system can quickly and specifically enter cancer cells to deliver the Cis chemotherapeutic agent and, with its pH-responsive behavior, can ensure fast release. The degree of Cis delivery and subsequent cytotoxicity in A549 cells as compared to normal human lung fibroblast cells (MRC-5) was investigated using a MTT assay (Supplementary Figure S4). For this, A549 and MRC-5 cell lines were treated with pristine Cis, nZIF-90, Cis⊂nZIF-90, and RGD@Cis⊂nZIF-90 in concentrations ranging from 0 to 100 µg ml^−1^ for 24 and 48 h. Based on cell viability (Figure 5), the cytotoxicity of RGD@Cis⊂nZIF-90 exhibited concentration-dependent behavior towards A549 cells after both the 24 and 48 h treatments as compared to nZIF-90 and Cis⊂nZIF-90. Noticeable cytotoxicity towards MRC-5 cells was evident at concentrations greater than 50 µg ml^−1^. At 6.25 µg ml^−1^, RGD@Cis⊂nZIF-90 induced 40 and 18% cell death in A549 and MRC-5 cells (*p* < 0.001), respectively, after 24 h. After 48 h, the cell death percentage (*p* < 0.001) significantly increased for A549 cells (65%), but not for MRC-5 cells (22%) when using the same concentration (6.25 µg ml^−1^) of RGD@Cis⊂nZIF-90. This demonstrated that there was better toxicity induction in A549 cells than in MRC-5 cells by the nZIF-90 delivery nanosystem. At a low concentration of 3.13 µg ml^−1^, RGD@Cis⊂nZIF-90 was classified as non-cytotoxic given that the cell viability was more than 80% in both A549 and MRC-5 cells after 24 and 48 h. Following this, Table 1 shows half-maximal (50%) inhibitory concentration (IC_50_) values for pristine Cis, nZIF-90, Cis⊂nZIF-90, and RGD@Cis⊂nZIF-90. In general, A549 cells achieved better IC_50_ values than MRC-5 cells after 24 and 48 h of treatment. Without the delivery nanosystem, pristine Cis had the lowest IC_50_ value with no difference being observed in either A549 or MRC-5 cells after 48 h treatment. Considerably better IC_50_ values of 8.79 ± 3.2 (24 h) and 6.01 ± 1.4 µg ml^−1^ (48 h) in A549 cells were calculated for RGD@Cis⊂nZIF-90 when compared to the values for Cis⊂nZIF-90 (32.45 ± 3.8 and 30.01 ± 3.0 µg ml^−1^ for 24 and 48 h, respectively). These values clearly demonstrate the effectiveness of the RGD@Cis⊂nZIF-90 delivery nanosystem in inhibiting tumor cell growth when compared to the RGD peptide-free Cis⊂nZIF-90 system. In comparison to A549 cells, RGD@Cis⊂nZIF-90 was less toxic towards MRC-5 cells with 31.07 ± 2.28 and 25.10 ± 3.1 µg ml^−1^ at 24 and 48 h, respectively.

**TABLE 1:** IC_50_ values for pristine Cis, nZIF-90, Cis⊂nZIF-90, and RGD@Cis⊂nZIF-90 against MRC-5 and A549.

Treatments	MRC-5 (μg ml^−1^)	A549 (μg ml^−1^)	Time (h)	Selectivity
				Index (SI)*^a^*
Pristine Cis	1.09 ± 0.2	1.820 ± 0.98	24	0.598
	0.945 ± 0.14	1.831 ± 1.09	48	0.516
nZIF-90	58.36 ± 2.6	57.37 ± 3.86	24	1.017
	46.63 ± 4.17	41.56 ± 2.66	48	1.122
Cis⊂nZIF-90	39.80 ± 2.97	32.45 ± 3.75	24	1.23
	34.71 ± 3.39	30.01 ± 2.97	48	1.157
RGD@Cis⊂nZIF-90	31.07 ± 2.75	8.79 ± 3.20	24	3.535
	25.10 ± 3.06	6.011 ± 1.38	48	4.176

^a^ SI = [IC_50(MRC-5)_/IC_50(A549)_].

Finally, we investigated the selectivity of pristine Cis, nZIF-90, Cis⊂nZIF-90, and RGD@Cis⊂nZIF-90 towards the A549 cells. Selectivity index (SI) is calculated as a ratio of the IC_50_ values for MRC-5 cells to the IC_50_ values for A549 cells with a value greater than 2 being regarded as significant and selective towards cancer cells. (Velozo-Sá et al., 2019) Remarkably, the SI values for RGD@Cis⊂nZIF-90 are 3.54 and 4.18 for its 24 and 48 h treatments, which provides conclusive evidence of its selectivity towards A549 cells (Table 1). In contrast, pristine Cis, nZIF-90, and Cis⊂nZIF-90 all had SI values of less than 2, from which minimal selectivity towards A549 cells was concluded. When taken together, the observed enhanced cytotoxicity and selectivity properties of RGD@Cis⊂nZIF-90 promotes its immense potential for use in active-targeting cancer therapy treatment.

**FIGURE 6:**
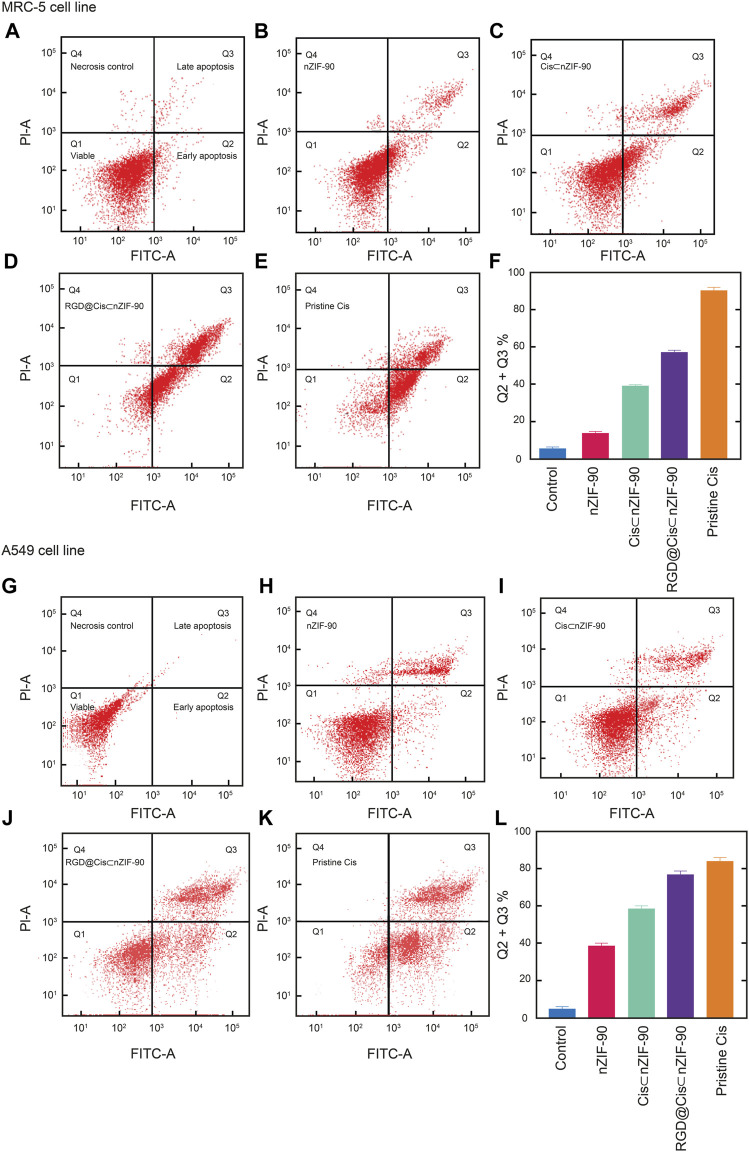
Apoptotic cell population obtained by flow cytometry analysis as presented using red dot plots of MRC-5 cells **(A–F)** and A549 cells **(G–L)**. After 48 h of treatment with the appropriate 50% inhibiting concentration (IC_50_ value) of nZIF-90, Cis⊂nZIF-90, RGD@Cis⊂nZIF-90 and Cis. The plots **(A)** and **(G)** show the control. A graphical representation of the total percentage of apoptotic cells for each is provided in **(F)** and **(L)**. The fluorescence intensity of Annexin V FITC and PI are used to identify Q_1_ as healthy cells (double negative), Q_2_ as early apoptotic (Annexin V positive), Q_3_ as late apoptotic/necrosis (double positive) and Q_4_ as necroptotic (double positive) cell populations. The percentage of apoptotic cells was calculated as the sum of Q_2_ + Q_3_. A minimum of 10,000 cells were counted and processed using software FlowJo_V10.7.2.

Flow cytometry analysis (Figure 6), with Annexin V/FITC and propidium iodide (PI) staining of A549 and MRC-5 cells, was employed to measure the apoptotic and necrotic cell populations upon treatment for 48 h with pristine Cis, nZIF-90, Cis⊂nZIF-90, and RGD@Cis⊂nZIF-90. The cells were considered as follows: 1) viable if negative for both Annexin V/FITC and PI staining (lower left quadrant; Q_1_); 2) early apoptotic if negative for PI and positive for Annexin V/FITC (lower right quadrant; Q_2_); 3) late apoptotic/necrotic if positive for both Annexin V/FITC and PI staining (upper right quadrant; Q_3_); or 4) necrotic if positive for PI and negative for Annexin V/FITC (upper left quadrant; Q_4_). The total percentage of apoptotic cells (Q_2_ + Q_3_) in A549 was higher than in MRC-5 when treated with Cis⊂nZIF-90 and RGD@Cis⊂nZIF-90. For both A549 and MRC-5 cell lines, the total number of apoptotic cells measured after treatment with pristine Cis was similar. The treatment of A549 cells with RGD@Cis⊂nZIF-90 led to a higher percentage of apoptotic cells (62%) than the treatments did with Cis⊂nZIF-90 (56%) or pristine Cis (60%). Furthermore, treatment with the RGD@Cis⊂nZIF-90 system produced a lower percentage of apoptotic cells (52%) in MRC-5 than pristine Cis (64%). Taken together, these results demonstrate that the RGD@Cis⊂nZIF-90 system induces more apoptosis in cancerous A549 cells than in non-cancerous MRC-5 cells due to the ability of its RGD homing peptide to target the A549 cells selectively and actively for delivering the Cis agent.

## Conclusion

In conclusion, nano-ZIF-90 was synthesized and loaded with a high amount of cisplatin (24 wt%) and its outer surface was post-synthetically modified with the cancer homing RGD peptide through covalent bond formation. The resulting delivery nanosystem, termed RGD@Cis⊂nZIF-90, exhibited satisfactory cellular uptake in cancerous A549 cell lines with exceptional pH-responsive drug release in conditions mimicking the A549 cellular environment. *In vitro* cytotoxicity measurements demonstrated that RGD@Cis⊂nZIF-90 was more successful in delivering Cis to A549 cancer cells with higher IC_50_ values and selectivity indices than normal MRC-5 cells. Apoptotic cells were also found to be more prevalent in the A549 cell population following treatment with RGD@Cis⊂nZIF-90. Based on the findings reported herein, we have demonstrated the successful synthesis of a nano-scale delivery system that is capable of actively and selectively targeting tumor cells to deliver chemotherapeutic drug under physiologically relevant environmental conditions.

## Data Availability

The original contributions presented in the study are included in the article/Supplementary Material, further inquiries can be directed to the corresponding authors.
