# Supplementary material for: ZIF-90 nanoparticles modified with a homing peptide for targeted delivery of cisplatin
Source: Front Chem. 2022 Dec 5;10:1076350. doi: 10.3389/fchem.2022.1076350 (PMC9760700; doi:10.3389/fchem.2022.1076350)
Supplement: Supplementary file 1 [file DataSheet1.PDF]

Supporting Information

ZIF-90 Nanoparticles Modified with a Homing Peptide for Targeted Delivery of Cisplatin

Adamu Abubakar,[1,2] Emilia Abdulmalek,*[,1] Wan Norhamidah Wan Ibrahim,[3] Kyle E. Cordova,*[,4,5,6] and Mohd Basyaruddin Abdul Rahman[1,5,6]

[1]Integrated Chemical BioPhysics Research, Faculty of Science, Universiti Putra Malaysia (UPM), 43400 UPM Serdang, Selangor, Malaysia

[2]Department of Chemical Sciences, Taraba State University, Jalingo P.M.B 1167, Taraba State, Nigeria

[3]Department of Biology, Faculty of Science, UPM, 43400 UPM Serdang, Selangor, Malaysia.

[4]Materials Discovery Research Unit, Advanced Research Centre, Royal Scientific Society, Amman 11941, Jordan

[5]Department of Chemistry, Faculty of Science, UPM, 43400 UPM Serdang, Selangor, Malaysia.

[6]Foundry of Reticular Materials for Sustainability (FORMS), Materials Synthesis and Characterization Laboratory, Institute of Advanced Technology, UPM, 43400 UPM Serdang, Selangor, Malaysia.

***Correspondence:** Kyle. E. Cordova (kyle.cordova@rss.jo) and Emilia Abdulmalek (emilia@upm.edu.my)

Quantification of the platinum using ICP-OES

The Cis loading level in ZIF-90@Cis was determined by inductively coupled plasma-optical emission spectroscopy (ICP-OES). The ICP-OES was utilized to determine the fraction of metal ions in complexes. A 10.958 mg portion of ZIF-90@Cis was digested in 10.0 mL of nitric acid to completely dissolve the Cis nanoparticles digested in the MOF-based matrix. Then, nitric acid (1.0 mL) with dissolved Cis was diluted with water (9.0 mL), and the resulting solution was used to measure the platinum concentration by ICP-OES. Calibration curves were prepared using a platinum solution of known concentration as a standard. From ICP-OES analysis, the Cis loading in ZIF-90@Cis was determined to be 24.03 wt%. Additionally, the steps involved in determining the amount of Cis in ZIF-90@Cis are given below. The concentration of Pt from ICP-OES gives C(Pt) = 73.0 ppb in the Cis from the sample that was analysed, and the dilution factor of 25/0.2 = 125 X in that sample. The real concentration of the digestion solution is thus 73 x 125 = 9125 ppb = 9.123 ppm (mg/L). The Pt came from the Cis sample and is contained in 50 mL. So, the total amount of Pt in 50 mL = 9.125 x 50/1000 = 0.456 mg.

0.456 mg came from 10.958 mg of Cis loaded in ZIF-90 sample,

thus, the mass % of Cis = 10.958/0.456 x 100 = 24.03 wt%.

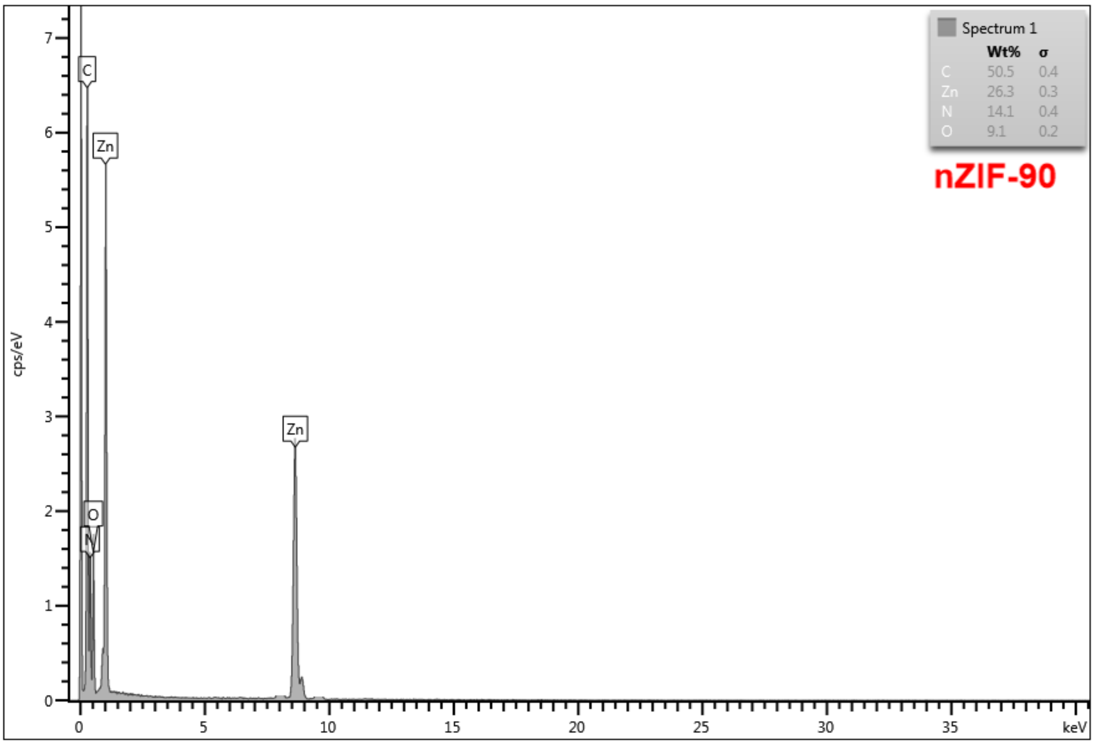

Figure S1. Energy dispersive X-ray (EDX) of nZIF-90 sample and quantitative result.

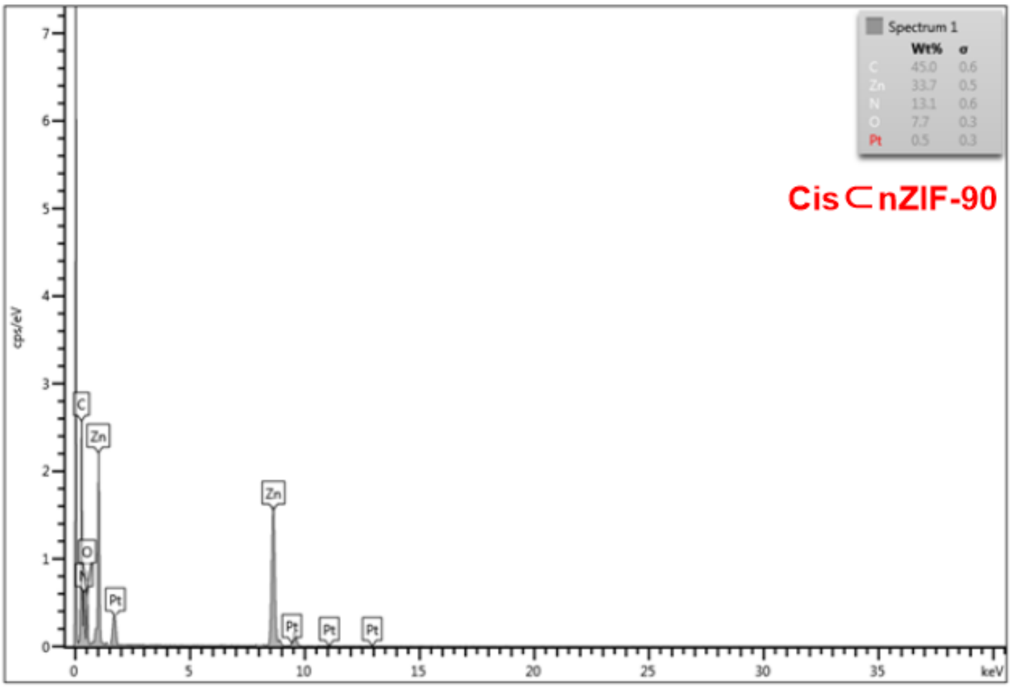

Figure S2. EDX of Cis⊂nZIF-90 sample and quantitative result.

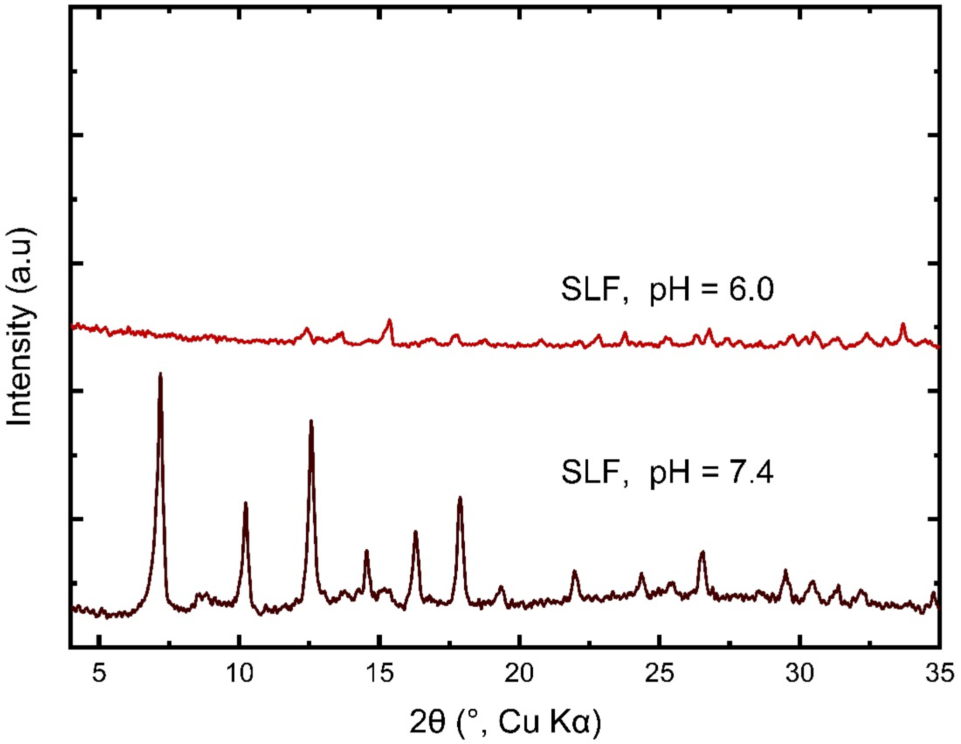

Figure S3. Powder X-ray diffraction (PXRD) pattern of RGD@Cis⊂nZIF-90 after24h incubation in SLF at pH 7.4 and pH 6.0

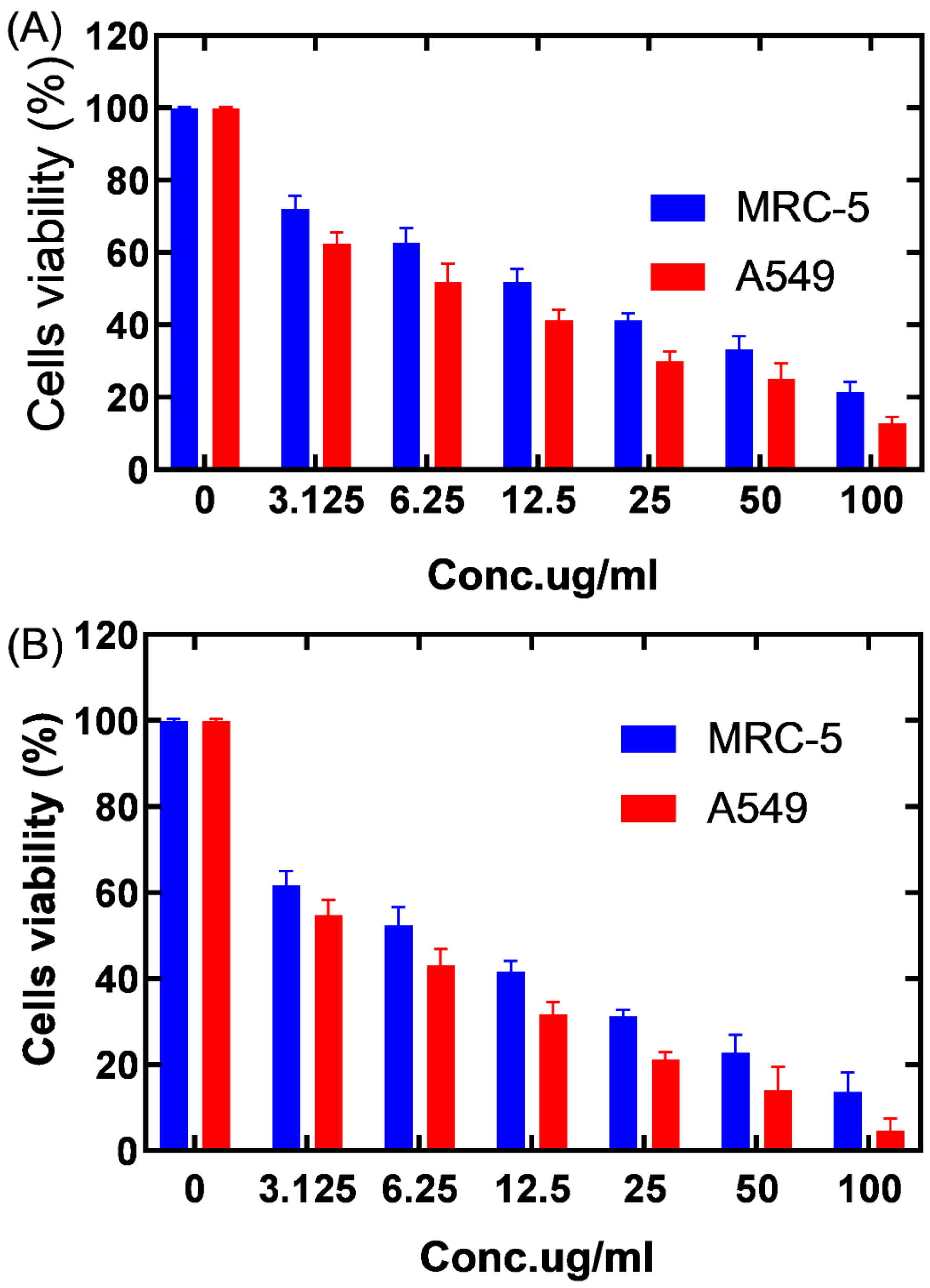

Figure S4. Cytotoxicity was assessed by MTT assay following treatment of A549 cells and MRC-5 cells with Cis for (A) 24 h and (B) 48 h. Percentages of cell viability are presented as a function of Cis concentration used
